# The impact of smart city construction on urban public health: evaluation using dual machine learning models

**DOI:** 10.3389/fpubh.2026.1796552

**Published:** 2026-03-20

**Authors:** Peizhi Xiong, Xiang Li, Hailong Yan

**Affiliations:** 1 School of Public Administration, South China University of Technology, Guangzhou, China; 2 School of Politics and Economic Management, Guizhou Minzu University, Guiyang, China; 3 School of Business Administration, Guizhou University of Finance and Economics, Guiyang, China

**Keywords:** dual machine learning, ecological environment, healthy human resource structure, innovative services, public health, smart city

## Abstract

**Introduction:**

Global public health is confronted with multiple challenges such as the frequent occurrence of infectious diseases, the increasing burden of chronic diseases, climate change, and the transformation of population structure. These social and environmental factors have exacerbated the complexity and difficulty of public health governance. Digital technologies represented by artificial intelligence and big data are developing rapidly, providing new tools and paradigms for addressing these challenges.

**Methods:**

This paper takes the pilot policies of smart cities in China as the standard natural experiment, and uses the panel data of 270 prefecture-level cities in China from 2007 to 2020. It empirically examines the impact of smart city construction (SCC) on public health level (PHL) by adopting a dual machine learning model.

**Results:**

The results show that SCC can significantly improve PHL, and the conclusion remains valid after robustness tests such as resetting the machine learning model, instrumental variable method, and multi-dimensional fixation. Mechanism tests show that SCC mainly enhances PHL by improving the quality of the ecological environment, enhancing the level of innovative services, and optimizing the structure of healthy human resources. Heterogeneity analysis revealed that the promoting effect of SCC on PHL was more pronounced in regions such as western China, large cities and key environmental protection cities.

**Discussion:**

The research conclusions can not only enrich the theoretical accumulation in the fields of smart cities and public health, but also provide replicable and scalable practical experience for other developing countries to enhance their public health governance capabilities through digital means under resource constraints.

## 1 Introduction

Public health is not only an important yardstick for measuring the quality of life of residents and social wellbeing, but also a fundamental condition for supporting the sustainable development of the economy and society. The acceleration of the global urbanization process, while reshaping the form of human residence, profoundly intensifies the complexity of public health governance (1). United Nations data shows that the proportion of urban population has jumped from 20% in 1950 to 45% in 2025, and it is expected that the urban population will increase significantly in the coming decades. Although urbanization brings about economic and service agglomeration effects, it is also accompanied by many health risks and inequality problems (2). On the one hand, the densely populated and highly mobile urban environment significantly increases the risk of infectious disease transmission. Non-communicable diseases such as cardiovascular diseases, diabetes, and cancer account for approximately the major proportion of global deaths, most of which are closely related to urban air pollution and unhealthy lifestyles (3). On the other hand, the global economic downturn has led to the superimposition of multiple risks such as economic vulnerability and social risks, profoundly affecting the level of healthy urban development. However, developing countries, due to their weak public health infrastructure, are facing more severe health problems. Therefore, it is particularly urgent to accelerate the establishment of a stable and long-term health investment guarantee mechanism. Meanwhile, digital technologies represented by artificial intelligence and big data are developing rapidly, providing unprecedented new tools and paradigms for addressing these challenges (4).

As an important product of the deep integration of the new round of technological revolution and urban governance, smart cities enhance the efficiency of urban refined management and service levels through digital transformation, create intelligent, healthy and sustainable urban environments, and can meet people's demands for high-quality health services (5). Meanwhile, as the world's largest developing country, China is typical in its government-led digital governance model, super-large-scale urban system, and rapidly advancing information infrastructure construction. Its smart city construction not only reflects the characteristics of leapfrog digital transformation in developing countries but also presents a unique path of parallel advancement of institutional coordination and technological integration. First of all, the construction of smart cities in China is promoted in a coordinated manner by the central government. Through the deep integration of the “Healthy China 2030” and “Digital China” strategies, an efficient policy transmission chain from top-level design to local innovation is formed. This strong mobilization model provides developing countries with a replicable state-led smart governance paradigm. Secondly, by 2025, China will have built the world's largest 5G network, with over 2.8 billion Internet of Things terminal users. Its real-time collection and intelligent analysis capabilities of medical and health data will rank first among developing countries. This enables China to fully unleash the health dividends of smart cities and avoid the common trap of disconnection from technology investment to application in developing countries (6). Thirdly, the scale effect formed by the construction of smart cities in over 500 cities has promoted the innovation of health governance models and the rapid diffusion of experience in public health emergency response systems (7). Therefore, research based on the Chinese sample not only provides a practical path for developing countries to avoid the technological trap of high investment and low coverage, but also offers beneficial experience for developed countries to bridge the digital divide in health.

From the existing literature, the research closely related to the topic of this article can be sorted out from two aspects. On the one hand, a large number of studies have examined the determinants of public health levels, which can be divided into micro and macro categories. At the micro level, personal socioeconomic characteristics such as income and education are widely regarded as key drivers of health outcomes (8, 9). At the macro level, environmental conditions and institutional backgrounds play equally important roles. Existing studies have confirmed that environmental regulations (10), air pollution (11), urbanization process (1), and energy poverty (12) have significant impacts on regional public health levels. These findings all indicate that public health is not only influenced by individual attributes but also deeply rooted in the broader urban governance system and infrastructure environment. The construction of smart cities, characterized by digital infrastructure, real-time data systems, and intelligent governance platforms, provides a new mechanism for improving public health governance. The construction of smart cities can enhance disease monitoring through real-time data collection, improve medical accessibility through digital service platforms, strengthen interdepartmental coordination through information sharing, and enhance emergency response capabilities through comprehensive monitoring systems.

On the other hand, existing research has conducted rich discussions on the development effects of smart cities, which can mainly be summarized into two dimensions: economic and environmental. In terms of economic development, the endogenous growth theory points out that economic growth mainly stems from the accumulation of technology and knowledge (13). SCC forms the synergy effect of technology-driven and factor aggregation by promoting the development of emerging technologies such as big data and artificial intelligence and the aggregation of high-end elements such as innovative capital and talents (14). It can promote scientific and technological innovation (15), optimize the efficiency of resource allocation (16), achieve industrial structure upgrading (17), enhance the level of specialization, and promote economic growth (18). In terms of ecological and environmental effects, the essence of SCC lies in the transformation of urban development models and the innovation of governance concepts. The traditional urbanization model, due to its excessive pursuit of scale expansion, has led to resource and environmental problems such as low energy efficiency, insufficient environmental carrying capacity, and intensified pollution (19). Through resource integration and digital governance innovation, SCC significantly reduces the intensity of environmental pollution (20), and promotes the transformation of cities to a low-carbon and efficient development model (21). Regarding the research on the relationship between smart cities and PHL, some scholars have adopted the differin-differences method and found that urban digital infrastructure has a positive impact on PHL (22, 44), while the research conclusion of Schram et al. (23) confirmed that digital infrastructure would instead exacerbate inequality in PHL.

Overall, SCC reshapes the urban governance model through digital means, achieving information integration, real-time monitoring, and cross-departmental collaboration, thereby comprehensively enhancing the operational efficiency and response capacity of the urban governance system. As a typical field of urban public governance, PHL has a natural interactive relationship with SCC. On the one hand, public health governance is highly dependent on the timeliness of information acquisition, the precision of resource allocation, and the multi-departmental collaboration mechanism, which are precisely the core manifestations of the improvement of digital governance capabilities in smart cities. On the other hand, the data infrastructure and intelligent decision-making platform formed by SCC also provide important support for disease monitoring and early warning, optimal allocation of medical resources, and public health emergency management. Therefore, from the perspective of modernizing urban governance, SCC may not only indirectly influence the level of public health through paths such as innovation effects, talent aggregation and environmental improvement, but also have a profound impact on PHL by reshaping the public health governance system.

Based on the above analysis, first, most of the literature only examines the single economic or environmental effect of SCC, and few literatures explore the theoretical logic and influence mechanism between smart cities and urban PHL from the perspective of urban PHL, and there is no consistent conclusion on the relationship between the two yet. Second, the existing methods for evaluating policy effects mostly adopt traditional causal inference models such as differin-differences and synthetic control methods. However, these methods often struggle to overcome problems such as the uncertainty of the form of the confusing factor function, dimension curse, and regularization bias, and thus cannot accurately identify the causal issues between SCC and PHL. Therefore, the question of this study is as follows: Can SCC increase PHL? If possible, through what channels does it function? Will the policy effects of SCC vary under different regions, city sizes and institutional conditions? To answer the above questions, this paper uses the urban panel data of 270 prefecture-level cities in China from 2007 to 2020 and, with the help of a dual machine learning model, empirically examines the impact of smart city pilot policies on urban PHL.

The main innovations of this article are as follows: Firstly, the socio-economic effects of SCC are identified from the perspective of PHL, and the mechanism of action is revealed based on three dimensions: ecological environment quality, innovative service level, and health workforce structure, providing empirical evidence for understanding the relationship between SCC and PHL. Second, a dual machine learning model is adopted for policy effect evaluation, effectively compensating for the biases caused by the presuppositions of traditional causal inference models and the “dimensional curse,” and scientifically extracting the improvement effect of SCC on PHL, making the research results more convincing. Thirdly, this study, by integrating regional characteristics, urban types, and the heterogeneity of environmental regulation intensity, clarifies the boundary conditions of the policy effects of SCC, and highlights the crucial role of SCC in addressing the shortcomings of traditional governance and enhancing local PHL, providing an important basis for optimizing the layout of smart city pilot projects and implementing precise policies.

## 2 Policy background and theoretical analysis

### 2.1 Policy background

Smart city refers to a new urban governance model that promotes the intelligence of urban planning, construction, management and public services by applying new-generation information technologies such as the Internet of Things, cloud computing, big data and spatial geographic information integration (24). Since IBM proposed the “Smart Planet” strategy in 2008, smart cities have gradually become an important policy tool for global cities to deal with rapid urbanization and the complexity of governance. Unlike the early focus on technology application and infrastructure upgrading, in recent years, the construction of smart cities in various countries has increasingly emphasized the use of digital means to enhance public governance capabilities and social service levels. In China, the construction of smart cities has gradually evolved from a simple information engineering project to a national-level digital governance strategy. In 2012, the Ministry of Housing and Urban-Rural Development launched the National Smart City Pilot program, aiming to promote the transformation of urban operation and management toward intelligence, refinement and collaboration by building a new generation of information infrastructure and urban data platforms. Subsequently, the construction of smart cities was incorporated into the national strategic systems such as “Digital China” and “Healthy China,” and its policy goals gradually expanded from the initial infrastructure construction to the fields of public service governance and social risk management. After the outbreak of the COVID-19 pandemic, the institutional connection between smart cities and public health governance has been further deepened. The digital health code system, the urban risk monitoring platform, and the epidemic prevention and control decision-making mechanism based on big data have gradually become important components of smart city infrastructure, significantly enhancing the efficiency of public health emergency response and the capacity for social risk governance.

Up to now, many regions in China have been actively promoting SCC and have initially established an urban governance framework covering multi-source data collection, real-time dynamic analysis, and intelligent decision support in multiple fields such as transportation, communities, and healthcare. This can provide strong computing power guarantee and technical support for the intelligent infectious disease monitoring and early warning system. Especially in the aspect of public health governance, the policy framework of smart cities continuously strengthens the institutional embedding of health goals. On the one hand, SCC promotes the digital transformation of medical and health services, enhancing the efficiency of medical resource allocation through the construction of electronic health records, telemedicine platforms, and smart hospital systems. On the other hand, SCC takes cross-domain data sharing and systematic collaborative governance as its core. Through the city's “smart brain” or big data bureau platform, it can achieve the departmental linkage and information integration required for infectious disease prevention and control. The smart city construction practices represented by Chengdu City in Sichuan Province, Chongqing City and Yichang City in Hubei Province show that the data aggregation mechanism based on the smart city basic platform can significantly enhance the convenience for disease prevention and control departments to obtain multi-source data, providing a referenceable practical model for establishing an efficient and collaborative infectious disease monitoring and early warning system.

This series of policy practices demonstrate that the construction of smart cities in China is no longer merely an important path for urban modernization, but has also become a significant institutional tool for promoting the digital transformation of public health governance. In 2025, the National Health Commission of China issued the “Implementation Opinions on Promoting and Regulating the Application and Development of ‘Artificial Intelligence + Medical and Health Care,”' which pointed out that by 2027, a number of high-quality datasets and trusted data Spaces in the health and medical industry should be established, and a number of vertical large models and intelligent agent applications for clinical specific diseases and specialties should be formed. Intelligent assistance for primary diagnosis and treatment, intelligent decision-making assistance for clinical specialized disease diagnosis and treatment, and intelligent services for patient visits have been widely applied in medical and health institutions. A number of national artificial intelligence application pilot bases in the medical and health field have been basically established, creating more high-value application scenarios and driving the high-quality development of the health industry. It is expected that by 2030, intelligent auxiliary applications in primary medical diagnosis and treatment will basically achieve full coverage. Efforts will be made to promote the widespread application of artificial intelligence technologies such as intelligent auxiliary diagnosis of medical imaging and intelligent auxiliary decision-making in clinical diagnosis and treatment in hospitals at or above the secondary level. The application standard and specification system of “artificial intelligence + medical and health care” will be basically improved, and a number of globally leading scientific and technological innovation and talent cultivation bases will be built. It can be foreseen that SCC will play an increasingly important role in the future urban public health governance process in China.

### 2.2 The impact of smart city construction on public health levels

Smart cities are essentially a high degree of integration of informatization and urbanization, representing the advanced stage of informatization development. SCC reconstructs the interaction paradigm between urban physical and digital Spaces by systematically integrating technologies such as the Internet of Things, big data, and artificial intelligence, exerting multi-dimensional empowering effects on PHL, mainly reflected in four aspects: risk early warning, resource allocation, service acquisition, and behavioral intervention. Firstly, the all-domain deployed intelligent perception network can break through the constraints of the traditional reliance on sampling statistics and post-event reporting, continuously capture biophysic signals such as the concentration of air pollutants and the transmission path of pathogens, form a dynamic health risk map, and shift disease prevention and control from passive response to active early warning (25). Secondly, at the resource allocation level, the cross-departmental data platform integrates multi-source heterogeneous information such as medical care, environmental protection, and transportation through algorithmic models to simulate the evolution path of public health emergencies, accurately calculate the distribution of resource demands, and optimize the emergency material dispatch and medical force deployment plans. Thereby significantly reducing problems such as prevention and control blind spots and response delays caused by information fragmentation (26).

Thirdly, intelligent technologies can optimize the spatio-temporal accessibility of health services. On the one hand, the remote diagnosis and treatment system weakens the spatial dependence of high-quality medical resources through high-definition image transmission and algorithm-assisted diagnosis. On the other hand, mobile medical applications achieve immediate responses to services such as chronic disease management and health consultation by breaking through the institutional barriers and time Windows of traditional service supply, thereby reducing the transaction costs of health access (27). Meanwhile, the real-time disclosure of environmental quality indices and epidemic risks can guide residents to take proactive protective measures and promote the shift of health behaviors from experience-driven to data-driven. Finally, it is worth noting that algorithmic bias may lead to vulnerable groups being excluded from the coverage of smart health services, while data security risks suppress residents' willingness to share medical information, constituting a new obstacle to health equity (28). However, from a systemic perspective, smart cities achieve sustainable development of urban PHL by deconstructing the bottlenecks of the asymmetry of health information (29), the spatio-temporal constraints of service acquisition, and the blindness of behavioral decision-making. Based on the above analysis, this paper proposes the following hypotheses:

**Hypothesis 1**. *SCC can significantly increase PHL*.

### 2.3 Mediating effect of SCC on PHL

#### 2.3.1 Mediating effect of ecological environment quality

The environmental exposure school holds that urban residents all live and work in specific environments, and environmental exposure, especially ecological and environmental pollution, is an important factor constituting potential threats and risks to public health. SCC provides a stable and lasting environmental support for public health improvement by guiding cities to transform toward greener, more intensive and efficient operation models.

On the one hand, SCC enhances the scientificity and controllability of ecological environment governance by strengthening the capabilities of environmental monitoring and information integration (30). Firstly, the environmental perception system constructed based on information technology can continuously track pollution sources and their evolution characteristics during the operation of a city, providing more timely and accurate information support for environmental governance. This information advantage helps to reduce blind spots and lag in environmental governance, enabling ecological and environmental governance to shift from extensive management to refined regulation, thereby promoting the continuous improvement of environmental quality. Secondly, SCC helps to form a more systematic and efficient environmental governance system by promoting information sharing and collaborative decision-making among departments, thereby reducing the negative impact of environmental pollution on residents' health (29). Digital governance means can enhance the transparency and traceability of environmental supervision, reduce institutional friction and implementation deviations in the governance process, and enable environmental governance goals to be more effectively transformed into actual improvement effects (31).

On the other hand, a good ecological environment helps to reduce pollution exposure and environmental risks, and forms constraints on the health conditions of residents. When the quality of the ecological environment improves, the adverse externalities to residents' health during the operation of cities weaken accordingly, and the pressure faced by the public health system also decreases accordingly. Meanwhile, the improvement of ecological environment quality helps to enhance the overall livability of cities, strengthen residents' sense of security and stable expectations for their living environment, and thereby promote the increase of PHL through long-term cumulative effects. Therefore, the following hypotheses are put forward:

**Hypothesis 2a**. *SCC can increase PHL by improving the quality of the ecological environment*.

#### 2.3.2 Mediating effect of innovative service levels

SCC strengthens the supply capacity and adaptability of innovative services by optimizing the allocation structure of public resources, guiding fiscal expenditure on science and technology to tilt toward areas related to public health, promoting the aggregation of innovative elements in the field of people's livelihood services (14). The core of innovative services lies in forming a systematic innovation system that covers technology research and development, service models, and management mechanisms. Firstly, in the field of medical services, innovative services such as the research and development of intelligent diagnosis and treatment equipment, the construction of remote medical platforms, and the development of cloud-based health data management systems supported by fiscal science and technology expenditures have broken down the barriers of uneven geographical distribution of medical resources, enhanced the accessibility and accuracy of medical services, and lowered the threshold for residents to receive medical treatment and health risks.

Secondly, in the field of public health governance, innovative services such as epidemic monitoring and early warning systems based on big data and artificial intelligence, as well as dynamic health risk assessment models, have strengthened the forward-looking prevention and control and rapid response capabilities of public health events, reducing the risk of infectious disease transmission and the scope of health damage. Meanwhile, innovative services such as digital health science popularization platforms and personalized health management apps supported by fiscal science and technology expenditures promote the precise dissemination of health knowledge and the scientific guidance of residents' health behaviors, reducing the occurrence probability of non-communicable diseases from the source. Finally, through the leverage effect of fiscal expenditure on science and technology (32), SCC can leverage social capital to participate in the supply of innovative services, form a diversified innovative service ecosystem.

**Hypothesis 2b**. *The level of innovative services plays a mediating role between SCC and PHL*.

#### 2.2.3 Mediating effect of the health workforce structure

The healthy human resource structure focuses on the allocation status and the degree of coordination among different types of human resources in the medical and health system. On the one hand, SCC reduces the information barriers among medical institutions at different levels by promoting the digitalization and platformization of medical service processes, which helps alleviate the long-standing problems of uneven distribution and structural imbalance of medical manpower (45). Meanwhile, SCC promotes the evolution of the medical service model toward greater specialization and collaboration by optimizing the public governance environment and service support system, thereby exerting a continuous impact on the health workforce structure (33). With the in-depth advancement of SCC, the demand for compound and specialized human resources in the medical and health system is constantly increasing. This change has driven the gradual optimization of the structure of health human resources in terms of quantity composition and functional division, laying a human resource foundation for the efficient operation of the medical service system.

On the other hand, the scientific allocation among different types of health manpower helps to strengthen the division of labor and collaboration within the medical service system, improve the coordination and continuity of the service supply process, and thereby enhance the overall efficiency of public health governance (34, 35). A reasonable human resource structure for health can reduce service bottlenecks caused by job misallocation and insufficient capabilities, and enable limited health resources to be more effectively transformed into health outputs. Meanwhile, the optimization of the health workforce structure helps enhance the public health system's response capacity to changes in health demands and strengthens the adaptability of medical services in the face of population structure adjustment and the evolution of health risks. A stable and reasonable human resource structure for health not only helps to improve the quality of medical services, but also enhances the resilience of the public health system, reduces the risks of service disruptions and system failures, and thus provides important support for the continuous improvement of PHL.

**Hypothesis 2c**. *SCC can enhance PHL by optimizing the structure of a healthy workforce*.

## 3 Research design

### 3.1 Model setting

At present, scholars mostly use the differin-differences (DID) method for policy evaluation, but the application of this method requires rather strict preconditions such as “parallel trends” and “random allocation.” Even when the preconditions are met, the control variables that vary over time can still cause the fixed effect estimators under the DID design to be biased. Compared with traditional causal inference models, dual machine learning models not only relax the assumptions about linear relationships, but also effectively control high-dimensional latent covariates (36). To accurately identify the impact of SCC policies on urban PHL, this paper adopts a dual machine learning model for causal inference.

The main reason lies in: (1) the establishment of smart city pilot zones is not completely random but is related to external conditions such as the economic development foundation and foreign trade potential of the region. Other external factors may affect the parallel trends of the experimental group and the control group, resulting in the DID model no longer being the optimal choice. (2) Dual machine learning models have unique advantages in variable screening and model estimation. By relying on diverse machine learning algorithms and their regularization techniques, they automatically carry out discrimination and screening work in a pre-determined set of high-dimensional control variables and obtain effective control variable combinations with high prediction accuracy. This model not only avoids the “dimension curse” caused by an excessive number of control variables, but also reduces the estimation bias resulting from the limited number of major control variables. (3) Non-linear relationships among variables can also lead to biased estimates of the results. The dual machine learning model can effectively handle the non-linear relationships between variables with the help of machine learning algorithms and improve the accuracy of causal recognition relationships. Based on this, this paper constructs the following dual machine learning causal inference model as follows:


$$\begin{array}{l} PHL_{it}=\theta_{0}SCC_{it}+g\left(X_{it}\right)+U_{it} \end{array}$$ (1)

$$\begin{array}{l} E\left(U_{it}|SCC_{it},X_{it}\right)=0 \end{array}$$ (2)

Among them, *i* and *t* represent the city and the year respectively; *PHL*_*it*_ refers to the level of public health in a city. *SCC*_*it*_ represents the pilot policy for smart cities. Its value is 1 in the year of the pilot and thereafter, and 0 otherwise. *X*_*it*_ is a set of high-dimensional control variables, which is a covariate that affects the explained variable through the function *g*(*X*_*it*_). Function *g*(*X*_*it*_) the specific form of the unknown, by machine learning methods learned that the estimator ĝ(*X*_*it*_), *U*_*it*_ as the random error term. θ_0_ represents the influence coefficient of the explanatory variable on the explained variable, with a conditional mean of 0. If the coefficient is positive, it indicates that SCC helps to improve PHL. If the coefficient is negative, it indicates that the city's PHL will be reduced.

Since dual machine learning must introduce a regularization term when estimating ĝ(*X*_*it*_) using machine learning and its regularization algorithm, although this can avoid excessive variance of the estimator, the estimation results it brings are biased. To accelerate the convergence speed, the orthogonal method is introduced to correct the bias, and the auxiliary regression is constructed as follows:


$$SCC_{it}=m(X_{it})+V_{it}$$ (3)

$$\begin{array}{l} E\left(V_{it}|X_{it}\right)=0 \end{array}$$ (4)

The specific operation process is as follows: Firstly, use a machine learning algorithm to estimate the auxiliary regression $\hat{m}(X_{it})$ and take the estimated value ${\hat{V}}_{it}=SCC_{it}-\hat{m}(X_{it})$ of its residuals; Secondly, a machine learning algorithm is adopted to estimate ĝ(*X*_*it*_), and the form of the principal regression model is changed to:


$$\begin{array}{l} PHL_{it}-ĝ\left(X_{it}\right)=\theta_{0}SCC_{it}+U_{it} \end{array}$$ (5)

Thirdly, using ${\hat{V}}_{it}$ as the instrumental variable of the explanatory variable *SCC*_*it*_ for regression, the following unbiased estimators were obtained:


$${\hat{\theta }}_{0}={\left(\frac{1}{n}\sum_{i\in I,t\in T}{\hat{V}}_{it}SCC_{it}\right)}^{-1}\frac{1}{n}\sum_{i\in I,t\in T}{\hat{V}}_{it}(PHL_{it}-\hat{g}(X_{it})$$ (6)

Finally, it is found that the convergence speed of $\sqrt{n}(\theta_{0}-{\hat{\theta }}_{0})$ toward 0 is faster, thereby obtaining an unbiased estimator of the disposal coefficient.

### 3.2 Variable definition

#### 3.2.1 Explained variable

Public Health level (PHL). The theory of the health production function holds that health is not a single outcome variable but a comprehensive output jointly “produced” by multiple input factors such as medical resource supply, institutional governance capacity, and social environmental conditions (37). Its realization process is reflected in the transformation mechanism from health input to health outcomes. Under this theoretical framework, the level of public health depends not only on the fundamental supporting conditions of the health system but also on the ultimate health outcomes and the level of risk control. Meanwhile, the framework of social determinants of health proposed by the WHO further emphasizes that the health status of the population is not only influenced by the supply of medical services, but also profoundly constrained by structural factors such as institutional capacity, resource accessibility, social environment and public governance systems. Therefore, the level of public health is essentially a comprehensive reflection of the capacity of the health system and the performance of health outcomes.

Based on the above theoretical analysis and drawing on relevant representative research results (38, 39), this paper constructs an index system from two complementary dimensions: public health foundation and public health performance. The former reflects the input capacity required to improve health, while the latter reflects the effectiveness of the results of health production. The integration of these two dimensions conforms to theoretical logic, that is, the improvement of public health depends on the coordinated enhancement of system capacity and health outcomes. When conducting specific measurement, the basic dimensions of public health reflect the investment capacity and institutional guarantee in the process of urban health production, including the government's health governance capacity, the level of medical resource allocation, and the supply status of medical and health care personnel, which are respectively measured by per capita fiscal medical and health expenditure, the number of medical and health institution beds per 10,000 people, and the number of doctors per 10,000 people. The dimension of public health performance reflects the outcome of healthy production, characterized from two aspects: health improvement and health loss, which are respectively measured by population survival rate and the incidence of urban infectious diseases. Finally, these indicators are measured by the entropy weight method.

#### 3.2.2 Explanatory variable

The core explanatory variable of this article is Smart City Construction (SCC). Regard the smart city pilot policy as a quasi-natural experiment. Set dummy variables based on whether a city is selected as a pilot city and in combination with the establishment time of the pilot city, that is, 1 after the establishment of the pilot city and 0 otherwise.

#### 3.2.3 Mechanism variables

Ecological environment quality (EEQ) Drawing on the practice of Xu et al. (40), indicators such as greenness, dryness, heat, and humidity were used for measurement. This study uses an improved pixel-based ecological environment quality model to assess the ecological environment quality in China. The specific calculation steps are as follows: Firstly, the Landsat data undergoes preprocessing including cloud removal, splicing, slicing, cropping, synthesis and resampling; Secondly, the greenness, dryness, heat and humidity indices were calculated based on the preprocessed annual average Landsat surface reflectance data, and the above four indices were calculated using MODIS data to interpolate the missing values of Landsat pixels. To ensure the reliability of the measurement results of ecological environment quality, this paper conducts accuracy verification in the process of data fusion and interpolation. Specifically, before interpolating the missing pixels of Landsat using MODIS data, complete pixel samples were randomly selected for simulated missing processing, and the same interpolation method was used for reconstruction. The interpolation accuracy was verified by calculating the root mean square error, the mean absolute error and the coefficient of determination. The results show that the interpolation errors of each index are all within the acceptable range of remote sensing ecological index research, indicating that the interpolation results have high reliability. Finally, the indicators were standardized, synthesized and principal component analyzed in sequence.

Innovation Service Level (ISL). The level of innovative services reflects the government's ability to support technological innovation and service supply through public investment. This study uses the proportion of urban fiscal expenditure on science and technology to measure the level of innovation services in cities, which can reflect the degree of attention and resource input intensity of local governments to innovation activities and the construction of innovation service systems. A higher proportion of fiscal expenditure on science and technology helps promote the transformation of technological achievements and innovation in public services, thereby providing important service support for the application of smart city construction to public health.

Health Workforce Structure (HWS). The structure of health human resources aims to depict the allocation characteristics of health human resources among different professional positions, rather than merely reflecting the scale level of human resources. A reasonable structure of health workforce is conducive to improving the efficiency and quality of medical service supply and reducing resource waste caused by human resource misallocation. This study represents the proportion of the total number of practicing physicians, registered nurses, and skilled workers among health technicians. This indicator measures the combined proportion of practicing physicians, registered nurses, and skilled workers among health technicians by incorporating them into a unified framework. It can more comprehensively reflect the degree of structural coordination between core service personnel and auxiliary support personnel in the medical service system.

#### 3.2.4 Control variables

To reduce the bias of omitted variables and obtain accurate policy effects, referring to existing studies (38, 39), this paper controls for the following factors that may affect urban PHL: (1) Educational attainment (ED). Measured by the average years of education; (2) Economic level (EL), measured by per capita GDP; (3) Social situation, represented by the number of registered unemployed people in urban areas at the end of the year; (4) The degree of openness is characterized by the ratio of the total urban passenger transport volume to the total urban population. (5) Environmental endowment (EN) is characterized by the green coverage rate of the built-up area. Finally, to characterize the possible non-linear relationships among the variables, this paper further introduces the quadratic term of the control variable and incorporates the urban fixed effect and the year fixed effect. The dimensions of the control variables thus formed have significantly increased, constituting a typical setting of high-dimensional control variables. In this situation, traditional regression methods are prone to overfitting problems. Therefore, this paper adopts a dual machine learning approach to flexibly handle high-dimensional control variables, thereby enhancing the robustness of the estimation results and the credibility of causal identification.

### 3.3 Data sources and descriptive statistics

Based on the availability and comparability of city-level data, this paper selects the panel data of 270 major cities in China from 2007 to 2020. The data on the ecological environment quality in this article is derived from the ecological environment quality dataset with a resolution of 500 m released by the National Earth System Science Data Center. The remaining indicator data are sourced from the “China Urban Statistical Yearbook,” the “China Health and Wellness Statistical Yearbook,” the Center for Socio-economic Data and Applications of Columbia University, and the EPS data Platform. For the problem of missing values in some indicators, interpolation method is adopted to make up for them. The descriptive statistics of the main variables are shown in Table 1. The mean value of the explained variable city PHL is 0.164, and the maximum and minimum values are 0.904 and 0.004, respectively. This indicates that there is an extreme differentiation in PHL among Chinese cities, and most cities are in a state of health poverty.

**Table 1:** Descriptive statistical results.

**Variables**	**Observations**	**Mean**	**SD**	**Min**	**Max**
*PHL*	3,466	0.164	0.095	0.004	0.904
*SCC*	3,466	0.171	0.376	0	1
*EEP*	3,466	0.489	0.129	0.101	0.812
*ISL*	3,466	10.133	34.945	0.053	550
*HWS*	3,466	0.794	0.043	0.614	0.914
*GDP*	3,466	10.510	0.697	4.595	13.056
*ED*	3,466	4.698	1.153	7.332	12.570
*OPEN*	3,466	42.833	174.947	0.064	8,233.640
*EN*	3,466	39.524	13.660	0.361	386.640
*SOCIAL*	3,466	26,782.130	33,688.883	142.672	600,501

## 4 Empirical results

### 4.1 Benchmark regression results

This paper adopts a dual machine learning approach to evaluate the impact of smart city pilot policies on urban PHL. Specifically, the samples are first randomly divided into training sub-samples and validation sub-samples, with a division ratio of 1:4. In each round of estimation, the training sub-samples are used to estimate the conditional expectation functions of the result variable and the treatment variable with respect to the control variable respectively by using the random forest algorithm, thereby obtaining the corresponding interference function. Based on this, this paper conducts the estimation of the interference function and the calculation of policy effects respectively based on different sample subsets to avoid overfitting problems when the machine learning model estimates the interference function and to satisfy the Neyman orthogonality condition. Subsequently, the policy effect is estimated by regression on the variables after residualization processing. In the model setting, this paper successively incorporates the primary and secondary terms of the control variables, and adds the fixed effects of the year and the city. The regression results are shown in Table 2. The results show that the regression coefficients of the smart city pilot projects are all positive at the 1% significance level, indicating that the smart city pilot policies can significantly enhance the PHL level of the city, thereby verifying Hypothesis 1.

**Table 2:** Benchmark regression results.

**Variables**	**(1)**	**(2)**	**(3)**	**(4)**
	***PHL***	***PHL***	***PHL***	***PHL***
*SCC*	0.074^***^	0.073^***^	0.081^***^	0.143^***^
	(2.71)	(2.65)	(2.99)	(3.38)
Constant term	0.013	0.012	0.013	0.006
	(1.19)	(1.18)	(1.49)	(0.70)
Control variables	YES	YES	YES	YES
Control variables (square term)	NO	YES	YES	YES
Year fixed effect	NO	NO	YES	YES
Urban fixed effect	NO	NO	NO	YES
*N*	3,466	3,466	3,466	3,466

^***^indicate that the regression results are significant at the levels of 1%, respectively. *t*-values are in parentheses.

### 4.2 Robustness test

#### 4.2.1 Tail reduction treatment of the research samples

Due to the existence of outliers in the research sample, which may cause bias in the estimation results, especially for some variables interpolated by interpolation methods, this paper performs two-sided tail-down processing on all continuous variables in the benchmark regression except for the core explanatory variable at 1% and 5% quantiles, and re-conducts the regression based on this. The regression results in columns (1) and (2) of Table 3 show that, considering the influence of outliers, the promoting effect of SCC on PHL always exists, and the benchmark regression results of this paper are robust.

**Table 3:** Robustness test I.

**Variables**	***PHL***	***PHL***	***PHL***	***PHL***
	**(1)**	**(2)**	**(3)**	**(4)**
*SCC*	0.153^***^	0.204^***^	0.093^***^	0.150^***^
	(3.64)	(5.27)	(2.68)	(3.53)
Constant term	0.002	−0.002	0.006	0.004
	(0.28)	(−0.30)	(0.73)	(0.49)
Control variables	YES	YES	YES	YES
Control variables (square term)	YES	YES	YES	YES
Year fixed effect	YES	YES	YES	YES
Urban fixed effect	YES	YES	YES	YES
*N*	3,466	3,466	3,466	3,466

^***^indicate that the regression results are significant at the levels of 1%, respectively. *t*-values are in parentheses.

#### 4.2.2 Multidimensional fixed effect

Although controlling the fixed effects of cities and years largely avoids mutual interference among cities, cities within the same province share similarities in policy environment, geographical location, and industrial development. Therefore, in this paper, the joint fixed effect of the provincial dummy variable and the time trend term is added to the benchmark regression to control the development trends of samples from different provinces in different years. The regression results are shown in column (3) of Table 3. The estimated coefficients of the core explanatory variables remain significantly positive, and the benchmark regression conclusion still holds.

#### 4.2.3 Eliminate the interference of relevant policies

The promoting effect of smart city pilot policies on urban PHL may be interfered with by other relevant policies of the same period during the evaluation process, resulting in empirical overestimation of the benchmark regression conclusion. Based on this, this article has controlled for policies that may cause interference, including the pilot program of Broadband China, the national-level big data comprehensive experimental zone, and the pilot program of innovative cities. Based on the benchmark regression, the above-mentioned policy dummy variables were controlled in sequence and in full by category. The specific regression results are shown in column (4) of Table 3. After excluding the influence of relevant policies in the same period, the effect of smart city pilot projects on enhancing the PHL of cities still exists.

#### 4.2.4 Instrumental variables

To alleviate the potential endogeneity problem, this paper further adopts the instrumental variable method to test the benchmark regression results. This paper selects the “number of post offices per million people” in each city in 1984 as the instrumental variable for the smart city pilot. This instrumental variable satisfies the two fundamental assumptions of correlation and exogeneity. Firstly, as an early communication infrastructure, the post office has profoundly influenced the spatial layout and development path of subsequent information and communication technologies, and may further affect whether a city will be included in the smart city pilot program earlier or more easily. The relevant conditions are met. Secondly, the above-mentioned historical data is nearly four decades ago and it is difficult to directly influence the current government governance efficiency through other channels, thus meeting the requirement of exogeneity. Given that the cross-sectional instrumental variables cannot be directly applied to the panel regression model, this paper interacts the above-mentioned historical variables with the time trend term to construct time-varying instrumental variables suitable for the panel structure and introduces them into the constructed partial linear instrumental variable model. The regression results obtained after the above processing are shown in column (1) of Table 4. After controlling for endogeneity, the smart city pilot still has a significant positive impact on PHL, further supporting the benchmark regression results of this paper.

**Table 4:** Robustness test II.

**Variables**	***PHL***	***PHL***	***PHL***	***PHL***
	**(1)**	**(2)**	**(3)**	**(4)**
*SCC*	0.228^***^	0.111^***^	0.201^***^	0.120^***^
	(2.76)	(2.87)	(4.49)	(5.00)
Constant term	0.066^***^	0.002	0.003	0.010
	(2.30)	(0.19)	(0.41)	(1.07)
Control variables	YES	YES	YES	YES
Control variables (square term)	YES	YES	YES	YES
Year fixed effect	YES	YES	YES	YES
Urban fixed effect	YES	YES	YES	YES
*N*	3,466	3,466	3,466	3,466

^***^indicate that the regression results are significant at the levels of 1%, respectively. *t*-values are in parentheses.

#### 4.2.5 Reset the dual machine learning model

To avoid estimation bias caused by parameter Settings and algorithm selection in dual machine learning models, this paper changes the sample segmentation ratio of dual machine learning from the previous 1:4 to 1:3 and 1:7, and replaces the random forest algorithm used for prediction with the gradient boost algorithm model (Gradboost). Gradient boosting algorithms can enhance prediction accuracy by iteratively optimizing the loss function and stably handle noise and outliers in the data to provide a lower standard error. The comparison result that the robust standard error corresponding to the smart city pilot in column (4) of Table 4 is lower than that of the benchmark regression confirms the advantages of this algorithm. However, the application advantages of the random forest algorithm in handling high-dimensional data, reducing the risk of overfitting, and providing easily interpretable results are more suitable for the research topic of this paper. Therefore, when the gap between the robust standard errors obtained by the two is not significant, this paper selects the random forest algorithm as the main prediction algorithm and the gradient boosting algorithm as a supplement for the robustness test. The regression results obtained after the above processing are shown in columns (2) to (4) of Table 4. The sample segmentation ratio of the dual machine learning model and the machine learning algorithm only change to a certain extent the size of the effect of the smart city pilot policy on improving PHL, and do not affect the conclusion that SCC can promote the PHL of the city.

### 4.3 Test of influence mechanism

Based on the theoretical analysis in the previous text, SCC can promote the development of urban PHL by improving the quality of the ecological environment, enhancing the level of innovative services and optimizing the structure of the health workforce. The regression results in column (1) of Table 5 show that the coefficient of SCC is significant at the 1% significance level, indicating that SCC has a significant promoting effect on the improvement of ecological environment quality. The results in column (2) of Table 5 show that the estimated coefficient of ecological environment quality is 0.143, passing the significance test at the 5% level. Therefore, SCC can promote PHL by improving the quality of the ecological environment, and Hypothesis 2a of this paper has been verified. Columns (3) and (4) of Table 5 report the regression results of the innovation service level as a mechanism. The coefficient value of the core explanatory variable SCC is 0.205, which is significant at the 1% level. The coefficient value of the innovation service level is significantly positive at the 5% level, indicating that SCC can promote public health development by improving the innovation service level of cities. Meanwhile, according to the estimation results in columns (5) and (6) of Table 5, the coefficients of SCC and the health workforce structure have both passed the significance level test of 1%, and the values are positive. Hypothesis 2c has been verified, that is, SCC can enhance the PHL of cities by optimizing the structure of the health workforce. To ensure the credibility of the mediating effect, this paper adopts Bootstrap sampling 1,000 times for further verification. The results show that the indirect effect values are all within the 95% confidence interval without zero, indicating that the mediating effect is statistically significant. In conclusion, SCC can not only directly improve PHL but also indirectly enhance it by improving environmental quality, strengthening innovative service capabilities, and optimizing the human capital structure.

**Table 5:** Mechanism test results.

**Variables**	**(1)**	**(2)**	**(3)**	**(4)**	**(5)**	**(6)**
	***EEQ***	***PHL***	***ISL***	***PHL***	***HWS***	***PHL***
*SCC*	0.028^***^	0.139^***^	0.205^***^	0.132^***^	0.773^***^	0.135^***^
	(3.97)	(3.21)	(2.84)	(3.39)	(3.01)	(3.22)
*EEQ*		0.143^**^				
		(2.38)				
*ISL*				0.054^**^		
				(2.50)		
*HWS*						0.010^***^
						(3.31)
Constant term	0.001	0.004	0.019	0.006	0.291	0.004
	(0.78)	(0.45)	(0.78)	(0.70)	(1.14)	(0.51)
Indirect effect	0.004		0.011		0.008	
95% CI	[0.000, 0.008]		[0.000, 0.023]		[0.001, 0.015]	
Control variables	YES	YES	YES	YES	YES	YES
Control variables (square term)	YES	YES	YES	YES	YES	YES
Year fixed effect	YES	YES	YES	YES	YES	YES
Urban fixed effect	YES	YES	YES	YES	YES	YES
*N*	3,466	3,466	3,466	3,466	3,466	3,466

^**^ and ^***^ indicate that the regression results are significant at the levels of 5% and 1%, respectively. *t*-values are in parentheses.

### 4.4 Results of heterogeneity analysis

#### 4.4.1 Heterogeneity analysis based on regional division

There are significant differences in economic development levels, population structures, technological stocks, industrial characteristics, etc. among different regions, which may lead to variations in the implementation effects of policies. Based on the geographical locations of the sample cities, this paper divided the samples into four groups: the eastern, central, western and northeastern regions, and conducted regression estimates respectively. The results are shown in columns (1) to (4) of Table 6. The coefficient values of the explanatory variables in the central and western regions were 0.119 and 0.251 respectively, both of which were significantly positive at the 1% level. However, the influence of SCC on PHL was not significant in the central and western regions. This differentiation stems from the fact that the original medical resources and environmental governance level in the eastern region are relatively high, and the marginal benefit of SCC is decreasing. Due to the scarcity of traditional medical resources and weak environmental supervision in the central and western regions, the remote services and real-time pollution monitoring of smart healthcare can more effectively fill the gap in public services. Especially in the western regions, the health benefits can be doubled by catching up through digital infrastructure. In the northeastern region, the rigid industrial structure and population outflow have weakened the efficiency of technology penetration, resulting in an insignificant effect.

**Table 6:** Heterogeneity analysis I.

**Variables**	***PHL***	***PHL***	***PHL***	***PHL***
	**(1)**	**(2)**	**(3)**	**(4)**
	**East**	**Central**	**West**	**Northeast**
*SCC*	−0.051	0.119^***^	0.251^***^	0.121
	(−0.56)	(2.59)	(3.74)	(1.46)
Constant term	−0.005	−0.003	0.156	−0.019
	(−0.29)	(−0.28)	(0.75)	(−1.06)
Control variables	YES	YES	YES	YES
Control variables (square term)	YES	YES	YES	YES
Year fixed effect	YES	YES	YES	YES
Urban fixed effect	YES	YES	YES	YES
*N*	1,128	1,009	855	474

^***^indicate that the regression results are significant at the levels of 1%, respectively. *t*-values are in parentheses.

#### 4.4.2 Heterogeneity analysis based on urban scale

With the continuous improvement of urbanization in China, there are differences in production input, technological level, etc. among cities of different scales, which leads to variations in the implementation effects of policies. Therefore, in this paper, the sample cities are divided into two groups: large cities and medium and small-sized cities, based on the “New Classification List of Chinese Cities” released by the New First-tier Cities Research Institute in 2025. This list is evaluated based on five dimensions: the concentration of commercial resources, the hub nature of the city, the activity level of urban residents, the diversity of lifestyles, and the potential for the future. The regression results are shown in columns (1) and (2) of Table 7. The estimated coefficient of the core explanatory variable in large cities is 0.046, but it is not significant. In medium and small cities, the coefficient value of SCC is significantly positive at the 1% level. The reason might lie in the fact that the original digital infrastructure and public health governance systems in large cities are relatively mature. SCC is more about system optimization and integration upgrades, and its marginal improvement in PHL is limited. However, small and medium-sized cities have significant room for improvement in terms of informatization level and governance capacity. SCC can significantly enhance the efficiency of their public health governance, thereby more effectively translating into improvements in health outcomes.

**Table 7:** Heterogeneity analysis II.

**Variables**	***PHL***	***PHL***	***PHL***	***PHL***
	**(1)**	**(2)**	**(3)**	**(4)**
	**Large cities**	**Medium and small cities**	**High environmental regulations**	**Low environmental regulation**
*SCC*	0.046	0.104^***^	0.235^***^	0.002
	(0.60)	(3.26)	(4.08)	(0.05)
Constant term	−0.008	0.004	−0.026	0.004
	(−0.49)	(0.46)	(-1.54)	(0.51)
Control variables	YES	YES	YES	YES
Control variables (square term)	YES	YES	YES	YES
Year fixed effect	YES	YES	YES	YES
Urban fixed effect	YES	YES	YES	YES
*N*	1,234	2,232	1,303	2,163

^***^indicate that the regression results are significant at the levels of 1%, respectively. *t*-values are in parentheses.

#### 4.4.3 Heterogeneity analysis based on the intensity of environmental regulations

Given that environmental factors are important exogenous conditions affecting PHL, this paper further divides the samples into cities with high environmental regulations and cities with low environmental regulations based on the list of key environmental protection cities published in the “China Environmental Yearbook,” in order to investigate the differentiated effects of SCC under the background of environmental governance. The grouped regression results of environmental regulation intensity in columns (3) and (4) of Table 7 show that SCC has a significant positive impact on PHL in cities with high environmental regulation, while the impact is not significant in cities with low environmental regulation. This indicates that stronger environmental regulations provide an important institutional basis for SCC to exert health effects. In cities with high environmental regulations, SCC can form synergy with existing environmental governance goals, further improving pollution exposure through strengthened environmental monitoring and precise governance, thereby significantly enhancing PHL. In cities with low environmental regulations, the environmental and health effects of SCC have not been fully released, and its health-promoting role is relatively limited.

#### 4.4.4 Heterogeneity analysis based on pilot batches

There are differences in the requirements for the cities applying for the national smart city pilot policies in different batches. That is, the different batches of the pilot policies not only have different impacts on the low-carbon development of cities in terms of time, but also imply the heterogeneous characteristics of individual cities and the differences in their impacts. This paper constructs dummy variables of different batches of smart city pilot projects for group regression to test the heterogeneity of policy pilot batches. The results are shown in Table 8. Only the influence coefficients of the second batch of pilot cities are significantly positive, while those of the first and third batches of pilot cities are positive but not significant. This might lie in the fact that the impact of smart city pilot policies on public health shows significant batch heterogeneity. The first batch of pilot projects are mainly in the stage of institutional exploration and infrastructure construction. The application of health governance is not yet mature, and the policy effects are difficult to be manifested in the short term. The second batch of pilot projects has entered the stage of application deepening based on the previous experience. The investment in smart healthcare and digitalization of public health has significantly increased, thereby generating obvious health improvement effects. The third batch of pilot projects was implemented relatively late, and the policy was still in its early stage of advancement. Moreover, due to the impact of the epidemic and the relatively weak regional basic conditions, its health effects were not significantly reflected.

**Table 8:** Heterogeneity analysis III.

**Variables**	***PHL***	***PHL***	***PHL***
	**(1)**	**(2)**	**(3)**
	**The first batch**	**The second batch**	**The third batch**
*SCC*	0.103	0.104^**^	0.084
	(1.40)	(2.04)	(1.36)
Constant term	0.001	−0.003	−0.009
	(0.12)	(−0.33)	(−0.91)
Control variables	YES	YES	YES
Control variables (square term)	YES	YES	YES
Year fixed effect	YES	YES	YES
Urban fixed effect	YES	YES	YES
*N*	2,752	2,821	2,608

^**^ indicate that the regression results are significant at the levels of 5%, respectively. *t*-values are in parentheses.

## 5 Discussions and conclusions

### 5.1 Discussions

In the face of a series of deep, complex and interrelated urban health risks caused by the rapid expansion of urban scale, how to improve the PHL of cities is an urgent topic to be studied. Therefore, this paper empirically investigated the impact of SCC on PHL using panel data of Chinese cities and obtained the following results. Firstly, the positive impact of SCC on PHL indicates that digital urban governance can enhance the efficiency of health services through information integration and resource synergy. This finding is consistent with the research conclusion of Xu et al. (41) on the improvement of residents' health levels by digital infrastructure. Meanwhile, it also echoes the viewpoint proposed by Schram et al. (23) of optimizing urban wellbeing through data-driven governance, indicating that the embedding of digital technology in the urban governance system is an important institutional tool for enhancing health equity. Secondly, ecological environment, innovative services and healthy human resources are the main channels for SCC to promote PHL, which echoes the research of Zhu et al. (42) on the spillover effect of digital transformation on environmental governance. Meanwhile, this result also supports Bentley's (43) view that the ecological environment is also a key condition determining the health level of residents, and smart cities provide a technical basis for the optimal allocation of these elements. Finally, SCC has demonstrated a stronger health promotion effect in the central and western regions, large cities, and key environmental protection cities, indicating that digital governance has a significant “marginal compensation effect.” This result also confirms the view that technological progress has differentiated governance performance under the background of unbalanced regional development, suggesting that SCC can to a certain extent narrow the gap in public health development.

The main research contributions of this paper are as follows: (1) Focusing on the specific policy practice of smart city construction, it expounds the impact effect of SCC on PHL, and analyzes its transmission mechanism from three aspects: ecological environment, innovative services, and healthy human resource structure, expanding the theoretical boundary of smart cities; Second, a dual machine learning model is adopted for policy effect assessment. The dual machine learning method, relying on the advantages of algorithms and data-driven modeling, is conducive to reducing the model setting bias caused by subjective factors and making the research results more reliable. Thirdly, this study, by integrating regional characteristics, urban types and the heterogeneity of environmental regulation intensity, clarifies the boundary conditions of the policy effects of smart city construction, providing an important basis for optimizing the layout of smart city pilot projects and implementing precise policies.

Although this article systematically examines the impact of SCC on PHL at the urban level, there are still certain limitations. Firstly, due to the limitation of data availability, PHL mainly uses macro indicators for measurement and has not yet delved into the individual health or micro-medical behavior level. Future research can conduct multi-level analysis in combination with the data from the residents' health survey. Secondly, this paper focuses on identifying mechanisms such as the environment, innovation and human resources, but pays insufficient attention to soft factors such as institutional quality and data sharing efficiency in digital governance. Subsequent research can further explore the moderating role of governance capacity in this regard. In addition, smart city policies have the characteristics of long-term nature and dynamic evolution. In the future, longer-term data or quasi-experimental dynamic methods can be adopted to examine the persistence and phased differences of their health effects.

### 5.2 Conclusions

This study utilized panel data from 270 cities in China from 2007 to 2020, and adopted a dual machine learning model to explore the impact of SCC on PHL and its mechanism of action. Additionally, a generalized random forest model was employed to investigate the heterogeneous influence of SCC on urban PHL. The empirical results show that: (1) The implementation of smart city policies has a significant promoting effect on PHL in the pilot areas. After a series of robustness tests including adjusting the research sample, eliminating relevant policy interferences, using the instrumental variable method, and resetting the dual machine learning model, the conclusion still holds. (2) Smart city pilot projects can promote the development of urban PHL by enhancing the quality of the ecological environment, improving regional innovation service levels, and optimizing a healthy human resource structure. (3) The promoting effect of SCC on PHL is more obvious in the central and western regions. In the eastern region, due to the higher level of original medical resources and environmental governance, the improvement effect brought by SCC is not obvious. Compared with large cities and non-environmental protection key cities, SCC has a significant promoting effect on the PHL of large cities and environmental protection key cities. Based on the above research conclusions, the following policy suggestions are put forward. The second batch of urban pilot projects can effectively increase PHL.

First, enhance cross-departmental data sharing and business collaboration, improve the ability to integrate and manage health information, and avoid redundant construction and resource dispersion. At the same time, we should increase support for the digital capacity building of grassroots public health, promote the downward flow of high-quality medical resources, and comprehensively enhance the accessibility and equalization of urban public health services. Second, the application of digital technology in ecological environment monitoring, pollution control and health risk early warning should be strengthened to enhance the intelligent level of urban environmental governance. At the same time, support the innovation of health-related technologies and the development of smart medical service models, and promote the digital integration of medical resources and the optimization of service processes. Relying on the smart platform, improve the training and allocation mechanism for public health talents and enhance the service capabilities of primary-level medical and health institutions. Third, increase support for the construction of smart health infrastructure and public health informatization in the central and western regions to narrow the gap in regional health development. In large cities and regions with strong ecological and environmental constraints, priority should be given to the layout of smart environmental protection and smart health collaborative systems to enhance the capacity for health risk monitoring and emergency response. For regions with a higher level of development, efforts should be made to upgrade smart health governance toward a refined and high-quality stage.

## Data Availability

The original contributions presented in the study are included in the article/Supplementary material, further inquiries can be directed to the corresponding author.
